# Supraglottic jet oxygenation and ventilation (SJOV) for resuscitation of injured soldiers and people in war field

**DOI:** 10.1186/s40779-022-00377-0

**Published:** 2022-04-12

**Authors:** Huafeng Wei

**Affiliations:** Department of Anesthesiology and Critical Care, Perelman School of Medicine, University of Pennsylvania, Philadelphia, PA 19104 USA

**Keywords:** Battlefield, War, Trauma, Acute injury, Respiratory depression, Hypoxia, Oxygenation, Resuscitation, Supraglottic jet oxygenation and ventilation (SJOV)

Dear Editor,

Soldiers or people in battlefield settings are frequently injured with acute trauma, resulting in respiratory depression, hypoxia and associated morbidity and mortality. Traumatic brain injury (TBI) is with as much as 80% to 90% of mild to moderate in combat casualties and contributed significantly to morbidity and mortality in battlefield settings. Correction of hypoxia at as capable as possible is expected to reduce or minimize the morbidity/mortality due to acute brain injury. An easy, quick and safe setup to promote oxygenation/ventilation of injured military personnel or peoples by non-medical personnel in battlefield settings immediately after acute injury before transfer to hospital is expected to reduce the morbidity/mortality due to injury mediated respiratory depression.

Supraglottic jet oxygenation and ventilation (SJOV) via use of WEI nasal jet tube (WNJ) has been demonstrated to promote oxygenation/ventilation in patients with respiratory depression or apnea due to propofol sedation/anesthesia with or without muscle relaxant, during elective or emergent difficult airway [1–5]. SJOV via WNJ was characterized by its simple setup, easy to learn and master, effective oxygenation with minimal side effects [2–4]. For acutely injured soldiers or peoples with respiratory depression in battlefield setting, following steps are proposed to promote oxygenation/ventilation immediately by non-medical personnel: (1) For injured people without loss of consciousness, measure distance from nose to ear using the markers on the outside wall of WNJ and insert the WNJ into the center of mouth to the measured distance or as deep as it can be tolerated, performing SJOV using a manual jet ventilator connected small oxygen tank (Additional file 1: Fig. S1) or oxygen bag with inside pressure reaching 20 psi. The working parameters for manual jet ventilator will be as followings: a fixed driving pressure of 20 psi, respiratory rate of 20/min and estimated inspiratory time 40% of each breathing cycle. The oral approach with its feature of less stimulation is usually tolerated well by conscious injured peoples, but with increased risk of WNJ position changes if not secured well. (2) For injured people with existing loss of consciousness or significantly decreased mental status, WNJ can be inserted into one of the noses for a measured distance from nose to ear and performing SJOV using above described setup (Additional file 1: Fig. S1), working parameters and method. The nose approach is prone to mild nose bleeding during insertion but has improved successes to place WNJ in correct position and can be secured well after correct placement [2–4].

Compared to regular nasal cannula, SJOV using WNJ via mouth promotes oxygenation/ventilation by synchronizing each high pressure driven oxygen jet pulse with inhaled gas into lung, higher fraction of inspired oxygen (FiO_2_) (see video at YouTube: https://www.youtube.com/watch?v=DXhfEMX5o6U). With WNJ inserted fully into mouth or nose, it is move invasive and needs some degree of sedation. Best oxygen/ventilation is usually achieved when the distal end of WNJ is located between vocal cord and uvula. SJOV via WNJ can be used to promote oxygenation/ventilation in awake patients with spontaneous breathing using low driving pressure (10–15 psi) and apnea patients with higher driving pressure (15–25 psi). A nasal cannula is less invasive and can be easily tolerated by awake injured people for prolonged use, but its ability to increase FiO_2_ and efficacy to promote oxygenation/ventilation is much less powerful than SJOV. Additionally, end-tidal carbon dioxide (PetCO_2_) can be easily monitored using WNJ, while most nasal cannulas are not equipped with capability of PetCO_2_ monitoring.

In conscious injured people for short period of use, SJOV has following advantages compared to other commonly used supraglottic oxygenation and ventilation devices: (1) Compared to a face oxygen mask, SJOV provides more efficient synchronized high pressure oxygen jet pulse into lung. (2) Compared to high flow oxygen nasal canula (HFNO), SJOV not only provides high FiO_2_ and promotes oxygenation, but also provides better ventilation to eliminate carbon dioxide [5]. For injured people with apnea, SJOV provides much more powerful unsynchronized oxygenation/ventilation than a face mask. It also provides more efficient ventilation than HFNO [5].

Considering the features of quick setup, easy to learn and practice even by non-medical personnel, minimal side effects of SJOV using WNJ, it has potential to be proposed as an effective measure to maintain oxygenation/ventilation in those acutely injured peoples in battlefield setting. Correction of hypoxia mediated morbidity/mortality as soon as acute injury of people in battlefield setting before transfer to hospital is expected to save soldiers and peoples during war time. The effectiveness and side effects of SJOV technique to save peoples in battlefield setting need to be studied and confirmed.

## Supplementary Information

**Additional file 1: Fig. S1.** Setup to generate supraglottic jet oxygenation and ventilation (SJOV) for battlefield setting. A small light and portable oxygen tank (or an oxygen bag) providing up to 20 psi pressure oxygen to a connected portable manual jet ventilator with its distal jetting end connected to the blue jet port of the WEI nasal jet tube (WNJ), capable of generating supraglottic jet oxygenation and ventilation (SJOV) quickly and safely in battlefield setting. The white port with distal end opening in the middle of WNJ lumen can be used to monitor end-tidal carbon dioxide pressure (PetCO_2_).

## Data Availability

Not applicable.

## Abbreviations

FiO_2_: Fraction of inspired oxygen
HFNO: High flow oxygen nasal canula
PetCO_2_: End-tidal carbon dioxide
SJOV: Supraglottic jet oxygenation and ventilation
TBI: Traumatic brain injury
WNJ: WEI nasal jet tube
