# Supplementary figures and images for: Supraglottic jet oxygenation and ventilation (SJOV) for resuscitation of injured soldiers and people in war field
Source: Mil Med Res. 2022 Apr 12;9:17. doi: 10.1186/s40779-022-00377-0 (PMC9003968; doi:10.1186/s40779-022-00377-0)

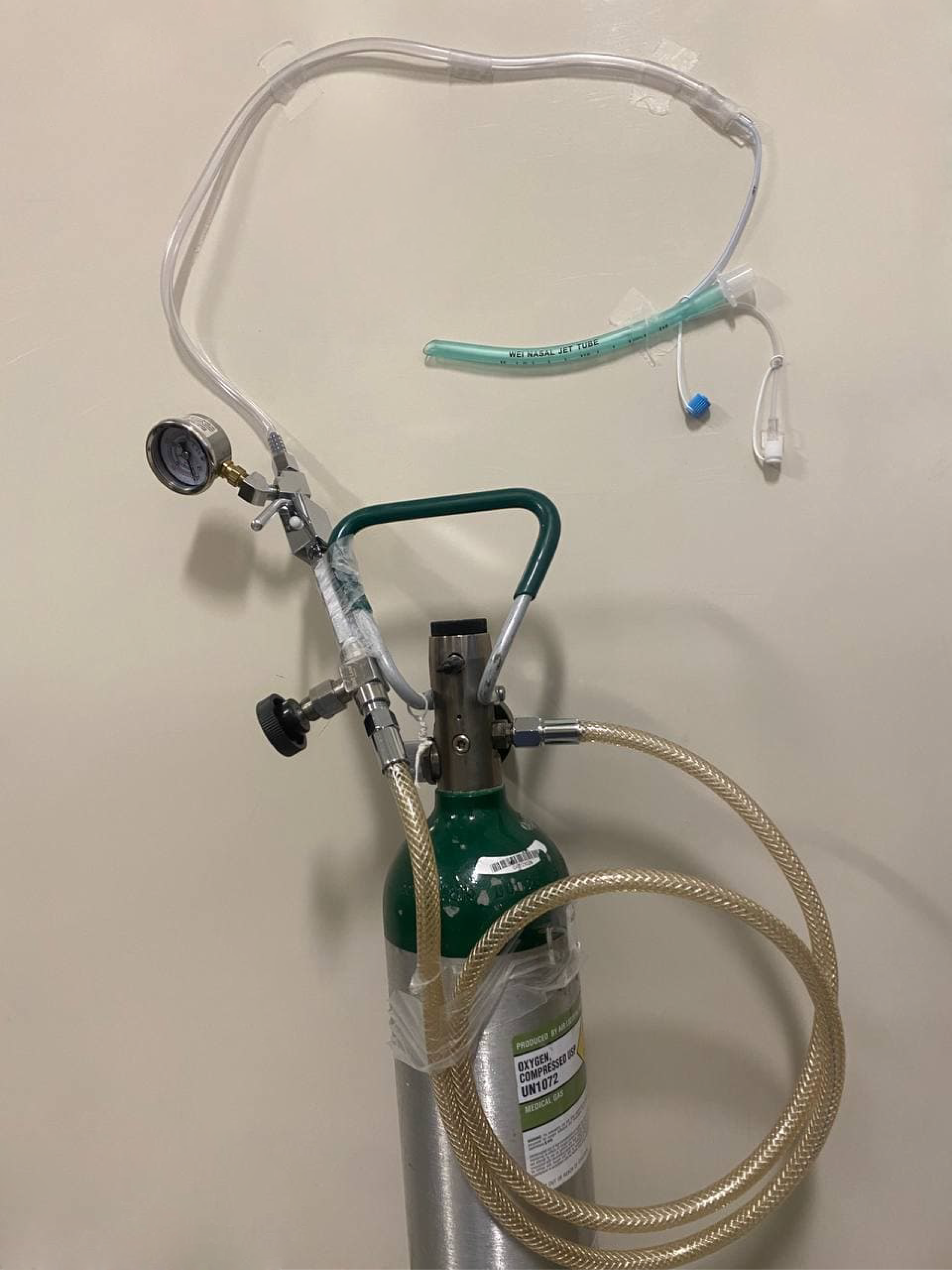

Supplement: Supplementary file 1 — Additional file 1: Fig. S1. Setup to generate supraglottic jet oxygenation and ventilation (SJOV) for battlefield setting. A small light and portable oxygen tank (or an oxygen bag) providing up to 20 psi pressure oxygen to a connected portable manual jet ventilator with its distal jetting end connected to the blue jet port of the WEI nasal jet tube (WNJ), capable of generating supraglottic jet oxygenation and ventilation (SJOV) quickly and safely in battlefield setting. The white port with distal end opening in the middle of WNJ lumen can be used to monitor end-tidal carbon dioxide pressure (PetCO2). [file 40779_2022_377_MOESM1_ESM.tif]
